# Supplementary material for: H(N3)dap (Hdap = 2,6-Diaminopurine) Recognition by Cu2(EGTA): Structure, Physical Properties, and Density Functional Theory Calculations of [Cu4(μ-EGTA)2(μ-H(N3)dap)2(H2O)2]·7H2O
Source: Molecules. 2023 Aug 26;28(17):6263. doi: 10.3390/molecules28176263 (PMC10488833; doi:10.3390/molecules28176263)
Supplement: Supplementary file 1 [file molecules-28-06263-s001.zip › molecules-2578657-supplementary.pdf]

Supplementary Material for manuscript:

H(N3)dap (Hdap = 2,6-Diaminopurine) Recognition by Cu_2(EGTA): Structure, Physical Properties, and Density Functional Theory Calculations of [Cu_4(μ-EGTA)$_2$(μ-H(N3)dap)$_2$(H_2O)$_2$]·7H_2O

Homa Mousavi, María Eugenia García-Rubiño, Duane Choquesillo-Lazarte, Alfonso Castiñeiras, Luis Lezama, Antonio Frontera and Juan Niclós-Gutiérrez*

Table of contents:

1. IR Figures Page 2

2. Pictures of the crystals Page 3

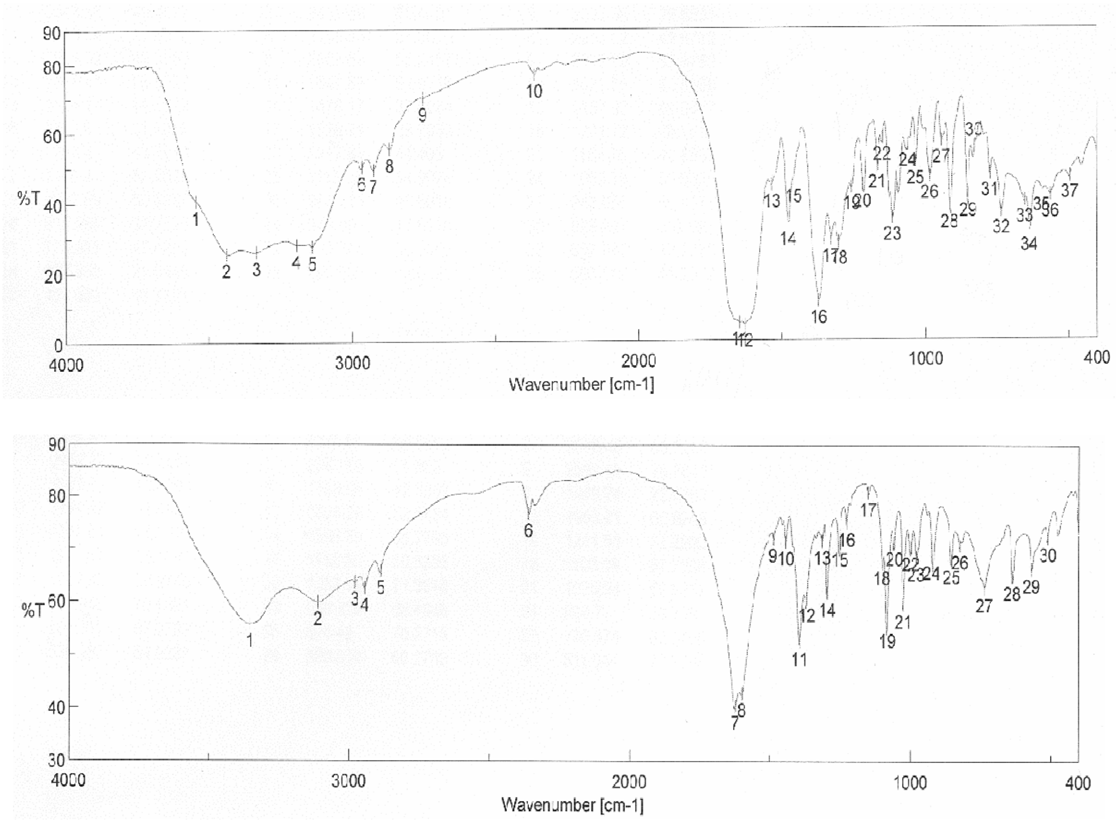

Figure S1. FT-IR spectra of the binary compound [Cu_2(μ-EGTA)(H_2O)$_2$]·$2H_2O$ (up) and the new ternary compound **1** (down).

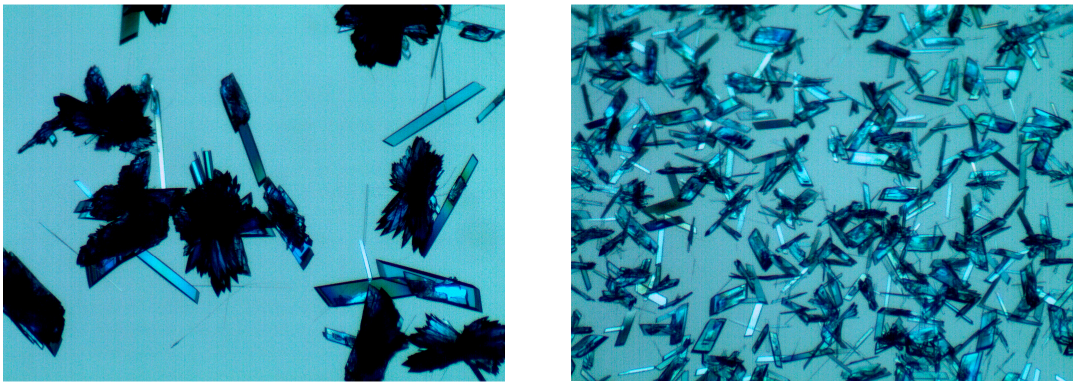

Figure S2. Photos of crystals of compound (**1**).
